# The evolutionary adaptation of wood‐decay macrofungi to host gymnosperms differs from that to host angiosperms

**DOI:** 10.1002/ece3.70019

**Published:** 2024-07-17

**Authors:** Xuetong Zhang, Yuran Dong, Yuying Li, Xiuping Wu, Siyu Chen, Mingyuan Wang, Yao Li, Zhiwei Ge, Min Zhang, Lingfeng Mao

**Affiliations:** ^1^ Laboratory of Biodiversity and Conservation, Co‐Innovation Center for Sustainable Forestry in Southern China, College of Ecology and Environment Nanjing Forestry University Nanjing China; ^2^ College of Life Science Nanjing Forestry University Nanjing China

**Keywords:** bipartite network, decompose, metabarcoding, phylogenetic tree, plant–fungi interaction

## Abstract

Wood‐decay macrofungi play a vital role in forest ecosystems by promoting nutrient cycling and soil structure, and their evolution is closely related to their host plants. This study investigates the potential evolutionary adaptation of wood‐decay macrofungi to their host plants, focusing on whether these relationships differ between gymnosperms and angiosperms. While previous research has suggested non‐random associations between specific fungi and plant deadwood, direct evidence of evolutionary adaptation has been lacking. Our study, conducted in a subtropical region, utilized metabarcoding techniques to identify deadwood species and associated fungi. We found significant evidence of evolutionary adaptation when considering all sampled species collectively. However, distinct patterns emerged when comparing angiosperms and gymnosperms: a significant evolutionary adaptation was observed of wood‐decay macrofungi to angiosperms, but not to gymnosperms. This variation may be due to the longer evolutionary history and more stable species interactions of gymnosperms, as indicated by a higher modularity coefficient (*r* = .452), suggesting greater specialization. In contrast, angiosperms, being evolutionarily younger, displayed less stable and more coevolving interactions with fungi, reflected in a lower modularity coefficient (*r* = .387). Our findings provide the first direct evidence of differential evolutionary adaptation dynamics of these fungi to angiosperms versus gymnosperms, enhancing our understanding of forest ecosystem carbon cycling and resource management.

## Graphical abstract

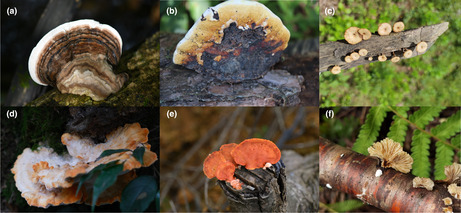

## 1 INTRODUCTION

**FIGURE 1:**
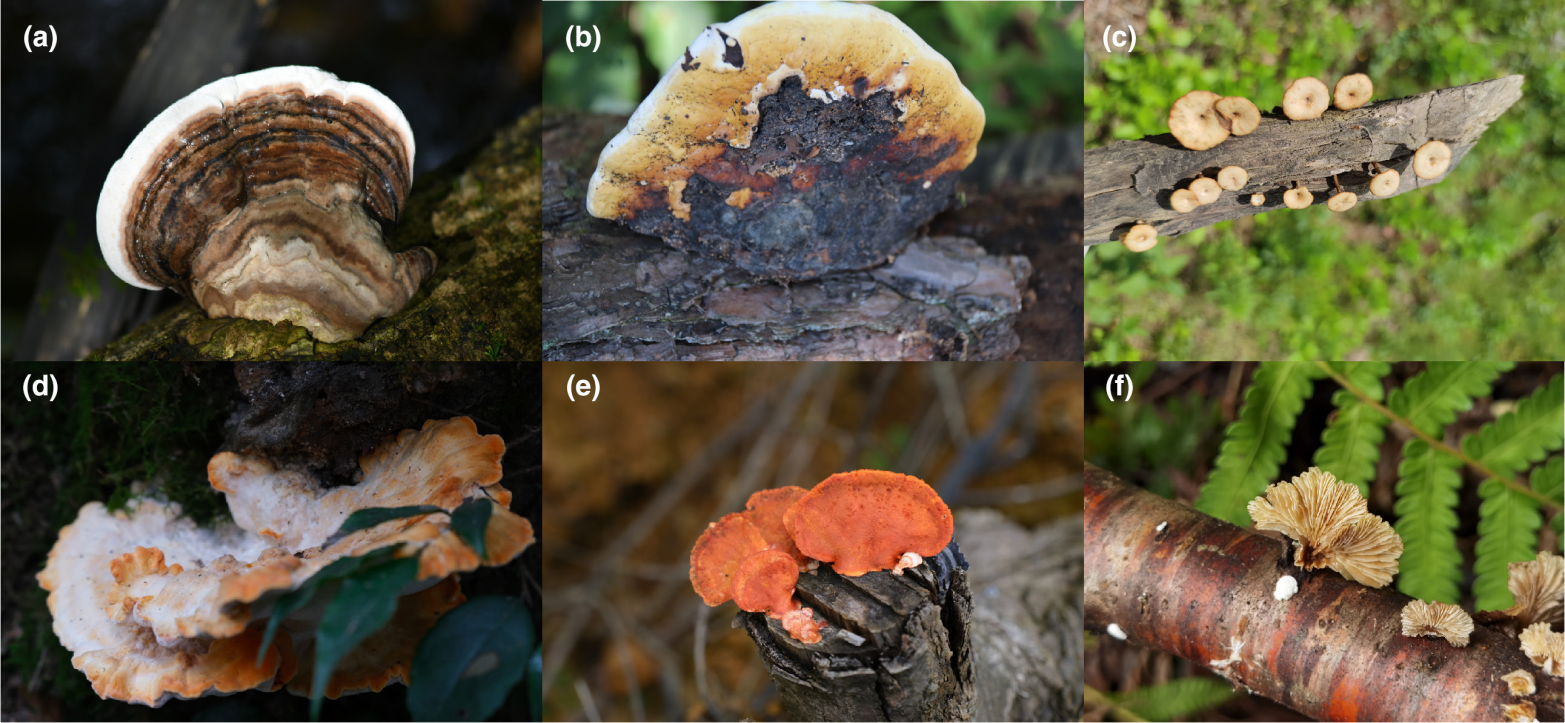
Selection of sampled wood‐decay macrofungi. (a). *Ganoderma applanatum* (b). *Fomitopsis pinicola* (c). *Lentinus arcularius* (d). *Laetiporus sulphureus* (e). *Trametes coccinea* (f). *Schizophyllum commune*.

Decomposition by wood‐decay macrofungi promotes carbon cycling within forest ecosystems (Pan et al., 2011; Rajala et al., 2012). Wood‐decay macrofungi (Figure 1) are fungi that can be observed with the naked eye and obtain nutrients via the decomposition of deadwood (Jönsson et al., 2008). Deadwood is a complex and low‐quality substrate composed of various simple polymer molecules that combine into several complex biopolymers, which creates a nutrient resource that is difficult for most organisms to access and decompose (Hoppe et al., 2016; Pan et al., 2011). Before the appearance of wood‐decay macrofungi, the decomposition of plant deadwood is accomplished primarily through insect feeding and fire (Bond & Scott, 2010). These decomposition methods may lead to outbreaks of insects or the destruction of tree resources, thus negatively affecting the balance of forest ecosystems. On the other hand, wood‐decay fungi mineralize or decompose most plant cell walls into simple bioavailable compounds using oxidases and hydrolytic enzymes (Floudas et al., 2012; Purahong et al., 2018). This decomposition method is relatively efficient and broadens the pathways for plant decomposition (Bond & Scott, 2010), positively promoting the flow of energy and carbon cycling in forest ecosystems.

Wood‐decay macrofungi decompose lignin, cellulose, and hemicellulose in deadwood by secreting oxidases and hydrolytic enzymes (Floudas et al., 2012). The amounts of lignin and cellulose in different plants vary due to their various functional traits (Huang et al., 2023). Different wood‐decay macrofungi secrete varying levels of hydrolytic enzymes and cellulases and employ multiple methods to decompose plants. White‐rot fungi extensively decompose lignin first before decomposing cellulose, with some white‐rot fungi, such as oyster mushrooms, possessing quite potent lignin degradation capabilities (Angel et al., 1994). Brown‐rot fungi decompose both cellulose and a small amount of lignin simultaneously. Additionally, some intermediate species possess characteristics of both white‐rot and brown‐rot fungi (Riley et al., 2014). Due to variations in lignin and cellulose contents in plant tissues and the varying decomposition abilities of wood‐decay macrofungi, different macrofungi show specific preferences when selecting plants, as demonstrated by experiments using next‐generation sequencing (Purahong et al., 2016). However, the lignin and cellulose contents in plant tissues can change as a result of their evolution (Reyt et al., 2021), and the types and quantities of enzymes secreted by wood‐decay macrofungi can also vary with their evolution (Alfaro et al., 2020). Both plants and wood‐decay macrofungi are constantly subjected to evolutionary pressures, and changes may even occur in their ecological roles over time (Scott, 1909). Despite this, wood‐decay macrofungi can maintain their preferences for specific plants even over long evolutionary timescales, indicating the likelihood of evolutionary adaptation of wood‐decay macrofungi to their host plants. In other words, the characteristics of wood‐decay macrofungi may have evolved along with the characteristics of plants. This hypothesis has not been explicitly tested.

The preferences of wood‐decay macrofungi for different plants are influenced by the lignin and cellulose contents of plant tissues. Lignin and cellulose contents can differ greatly between gymnosperms and angiosperms (Bonawitz & Chapple, 2010). Lignin is a hydrophobic and heterogeneous biopolymer crucial for plant tissue rigidity (Barros et al., 2015; Coleman et al., 2008). Overall, gymnosperms are taller and have stiffer plant bodies as compared to angiosperms, and lignin content is typically higher in gymnosperms (Berendse & Scheffer, 2009; Cornwell et al., 2008). Not surprisingly, the species of wood‐decay macrofungi that occur on gymnosperms and angiosperms differ (Purahong et al., 2016). Furthermore, some studies have shown that gymnosperms are generally more resistant to decomposition from wood‐decay macrofungi than angiosperms (Baber et al., 2016). Gymnosperms have difficulty utilizing nutrients in soil litter, and their litter is also more resistant to decomposition. On the other hand, angiosperms have faster growth rates, require greater nutrient supplies, and promote nutrient release in the soil through the production of easily decomposable litter (Berendse & Scheffer, 2009), leading to a faster life cycle. Angiosperms have a higher interaction frequency with wood‐decay macrofungi than gymnosperms, increasing the probability of evolutionary adaptation of wood‐decay macrofungi to angiosperms. Additionally, gymnosperms are evolutionarily older than angiosperms. Prominent gymnosperm families, such as Cheirolepidiaceae (Alvin, 1982), were the dominant tree species in forests long before the Cretaceous period (Crane et al., 1995; Doyle, 2001; Friis et al., 2006), while angiosperms were relatively rare during that time and only experienced a significant ecological advantage later (Wolfe, 1997). In the Late Cretaceous, angiosperms began to dominate the forest ecosystem, and this coincided with the rapid diversification of wood‐decay macrofungi (Berendse & Scheffer, 2009). This temporal coincidence suggests a possible mutual influence between the evolution of angiosperms and wood‐decay macrofungi. However, whether there are differences between the evolutionary adaptation of wood‐decay macrofungi to gymnosperms and the evolutionary adaptation of wood‐decay macrofungi to angiosperms has yet to be addressed rigorously.

In this study, we investigate the evolutionary adaptation of wood‐decay macrofungi to their host plants. We used metabarcoding and morphologic observation to identify the species of host plants and their wood‐decay macrofungi. We then used the “test of host‐parasite coevolution” function in R to characterize the evolutionary adaptation of wood‐decay macrofungi to their host plants. The main objectives of our research were twofold: (i) to determine whether evolutionary adaptation occurred of wood‐decay macrofungi to their host plants, and (ii) to investigate whether evolutionary adaptation of wood‐decay macrofungi to gymnosperms was similar to evolutionary adaptation of wood‐decay macrofungi to angiosperms. To address these two questions, we aimed to characterize the mechanisms of evolutionary adaptation of wood‐decay macrofungi to their host plants and to elucidate the reasons for any differences in the evolutionary adaptation of wood‐decay macrofungi to gymnosperms versus angiosperms. Ultimately, our study seeks to provide a theoretical basis for wood decomposition and carbon cycling in forest ecosystems.

## 2 MATERIALS AND METHODS

### 2.1 Sampling area

Our study area was located in subtropical regions, with the main sampling sites located in Chun'an County, Zhejiang Province, China (29°11′–30°02′ N, 118°20′–119°20′ E). Samples of wood‐decay macrofungi and their host plants were collected from September 2021 to May 2022. Survey areas with good ecological conditions and high coverage of forest vegetation were prioritized over areas heavily impacted by human activities.

### 2.2 Construction of plant and wood‐decay macrofungi network

We investigated the bipartite network of wood‐decaying macrofungi and their host plants. For wood‐decay macrofungi, we used a two‐step method for species identification. Firstly, we identified species of wood‐decay macrofungi collected directly from their host plants based on morphological characteristics. However, this method cannot detect macrofungi that fruit outside the sampling periods. To include additional macrofungi that were present on the same wood but not visible at the time of collection, we employed a metabarcoding technique for more comprehensive species detection. The specific operation was as follows: we collected dead wood in which wood‐decay macrofungi occur, and dissect a piece from central of the dead wood with sterilized scalpels in a sterile environment. The obtained samples were then identified using metabarcoding techniques. Deadwood DNA was extracted using TIANGEN Plant Genomic DNA Kit (DP‐305). In the process of PCR amplification, the primers used for wood‐decay macrofungi are ITS5 and ITS2 (White et al., 1990). We carefully excluded any organisms not related to the study's focus, such as soil macrofungi and other non‐wood‐decay fungi, ensuring that only wood‐decay macrofungi were retained for analysis.

We used metabarcoding to identify the species of dead wood, too. We used primer pairs Z1aF and hp2R of rbcl to amplify the deadwood DNA extracted above (Hofreiter et al., 2000). Initially, we attempted to identify the host plant using the Sanger sequencing method; however, the sequence quality was low. Therefore, we identify host plants through metabarcoding techniques, which provide better sequence quality in our preliminary experiments.

### 2.3 Reconstruction of phylogenetic trees for host plants and wood‐decay macrofungi

We reconstructed phylogenetic trees for both wood‐decay macrofungi and host plants. The phylogenetic tree for host plants was reconstructed using V.phylomaker (Jin & Qian, 2019). We used the Tidyverse package in R to clean the data and the Picante package to visualize the species extracted from the backbone tree as a phylogenetic tree. We using the same method reconstructed phylogenetic trees for gymnosperms and angiosperms. These phylogenetic trees were later used to test for evolutionary adaptation of wood‐decay macrofungi to their host plants.

Past research into the phylogenetic relationships of macrofungi has been limited, with only a few studies focusing on the phylogenetic analysis of macrofungi, and the number of species included in those analyses was insufficient for this study (Li, Steenwyk, et al., 2021; Olito & Fox, 2015; Varga et al., 2019). Therefore, to obtain a phylogenetic tree of wood‐decay macrofungi, we incorporated species encountered in our study into the fungal phylogenetic tree of Agaricomycetes reconstructed by Marisol et al. (2020). If a species encountered among our samples was included in Marisol's tree, no modifications were made to the phylogeny. If a species encountered among our samples was not included in Marisol's tree, it was treated as a randomly selected species from the same genus. If the genus was not included in Marisol's tree, it was treated as a randomly selected species from the same family. This process was repeated three times. Then, using the “phytools” package and “paleotree” package in R (Bapst, 2014; Revell, 2012), we set a minimum branch length to reconstruct a new binary, ultrametric phylogenetic tree for wood‐decay macrofungi. Using the same method, we reconstructed phylogenetic trees for wood‐decay macrofungi associated with gymnosperms and angiosperms.

### 2.4 Testing the hypothesis of evolutionary adaptation of wood‐decay macrofungi to host plants

We manually identified associations between wood‐decay macrofungi and their host plants. These data were converted into a 0–1 matrix using the reshape2 package (Olito & Fox, 2015), with plants represented as columns and macrofungi represented as rows. We generated separate 0–1 matrices for all host plants with their wood‐decay macrofungi, host gymnosperms with their wood‐decay macrofungi, and host angiosperms with their wood‐decay macrofungi. In the matrix, “1” means that the wood‐decay macrofungi can occur on the plant, and “0” means that the wood‐decay macrofungi cannot occur on the plant. Using the “parafit” function in R (Legendre et al., 2002), a package that tests for coevolution between host and parasite phylogenetic branches, we tested the hypothesis of evolutionary adaptation of wood‐decay macrofungi to their host plants. The null hypothesis of the global test was that the evolution of the two populations was independent, as supported by the two phylogenetic trees and the host–parasite association matrix. In the case of evolutionary adaptation, closely related wood‐decay macrofungi would occur on closely related host plant groups, with the results of the “parafit” test significantly deviating from the null model (*p* < .05).

### 2.5 Statistical analysis of the bipartite network structure and corresponding relationships between wood‐decay macrofungi and host plants

We calculated the average number of links between wood‐decay macrofungi and their host plants. Then, we performed *F*‐tests using these averages to investigate the differences in the evolutionary adaptation of wood‐decay macrofungi to both gymnosperms and angiosperms. A significant *F*‐test (*p* < .05) would indicate a difference in the dispersion of links between gymnosperms and their wood‐decay macrofungi as compared to angiosperms and their wood‐decay macrofungi.

We used the bipartite package in R to perform these analyses and calculate the related parameters. The bipartite package allows for the analysis and computation of matrix networks and the calculation of various ecological network structures and descriptive indices. This package focuses on networks consisting of only two trophic levels and allows for quantification and visualization of network structure and topology. Our study used the computeModules function from the bipartite package to calculate the modularity (Dormann & Strauss, 2013). Modularity was quantified with Newman's metric using the DIRTLPAwb+ algorithm, we set this algorithm to 10^6^ steps to search for the highest modularity (Beckett, 2016; Dormann & Strauss, 2014). As it is an optimization algorithm, the highest values found may vary among runs, so we repeated the analysis five times for each network and accepted the highest modularity obtained (Vizentin‐Bugoni et al., 2016). The modularity ranges from 0 to 1, with higher values indicating more robust modularity and close specialization between plants and their wood‐decay macrofungi. To test the significance of modularity, we compared metric values from observed matrices with metric values obtained from random matrices created using the vaznull null model from the R package bipartite (Dormann et al., 2009). This null model reshuffles interactions in a matrix of the same size, connectance, and marginal totals of the observed matrix. We created 100 null matrices to test for modularity because is a time‐consuming optimization algorithm (Vizentin‐Bugoni et al., 2019). For consistency, for each of the 100 null matrices, we calculated modularity five times and kept the highest values, as we did for the observed network. We considered a metric of modularity to deviate significantly from a random structural pattern when the observed value was higher than the confidence interval (95%) obtained from null matrices (Li et al., 2022; Vizentin‐Bugoni et al., 2019).

We also counted the numbers and proportions of wood‐decay macrofungi on different plants to provide insight into the evolutionary adaptation of wood‐decay macrofungi to their host plants.

## 3 RESULTS

### 3.1 Evolutionary adaptation of wood‐decay macrofungi to host plants

**FIGURE 2:**
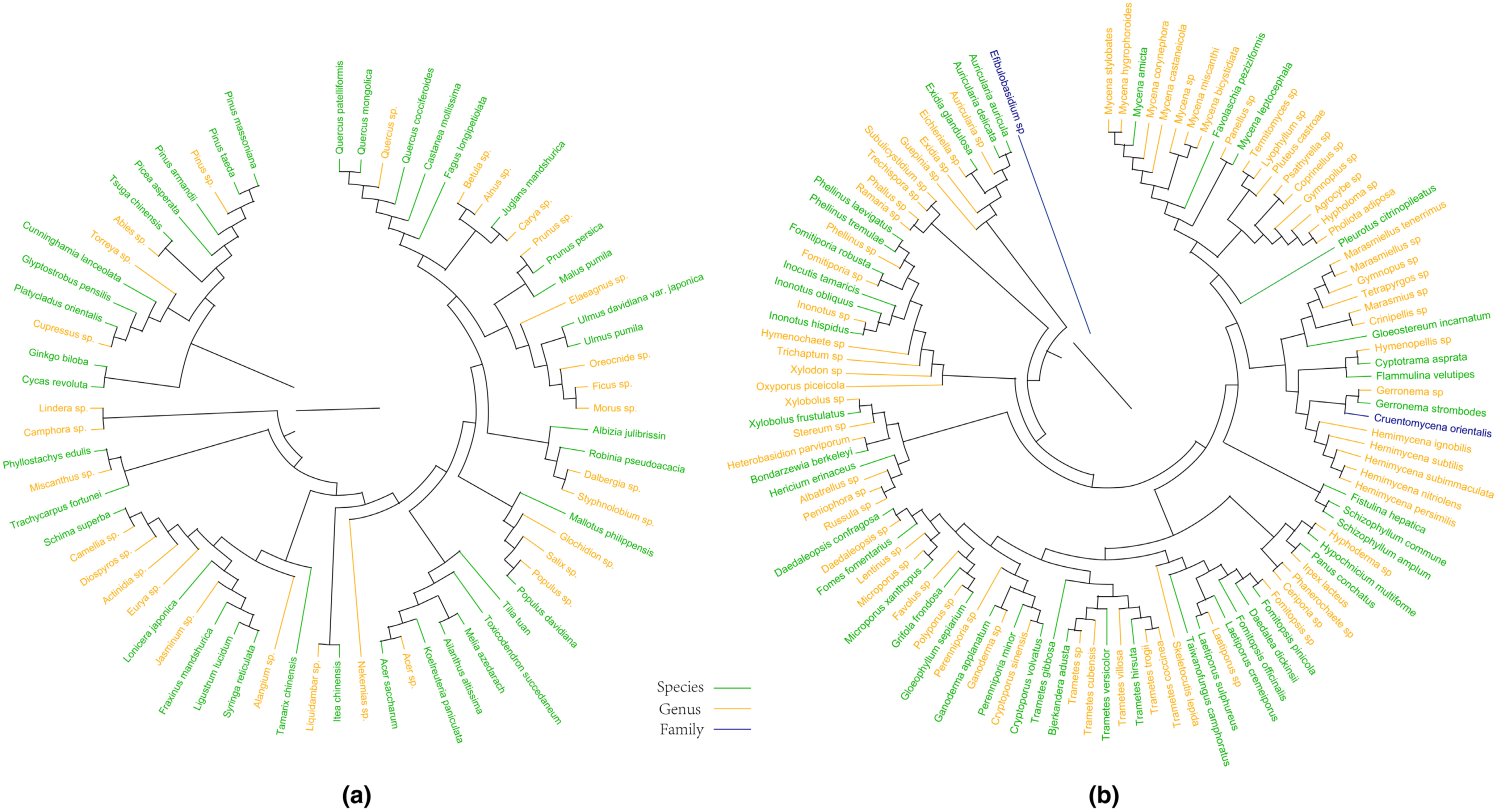
Phylogenetic Trees of Host Plants (a) and Wood‐decay macrofungi (b). The green samples were identified at the species level, the yellow samples were identified at the genus level, and the blue samples were identified at the family level.

We identified 116 species of wood‐decay macrofungi belonging to 41 families and 76 genera. These macrofungi were found on 69 different species of host plants belonging to 36 families and 59 genera. Despite using precise metabarcoding identification methods for plants and macrofungi, we were unable to identify all samples at the species level. Only 39 host plants were identified at the species level, while the remaining 30 were identified at the genus level. The phylogenetic relationships among host plants are shown in Figure 2a. As for wood‐decay macrofungi, 43 wood‐decay macrofungi were identified at the species level, 71 were identified at the genus level, and the remaining two wood‐decay macrofungi were identified at the family level. Their phylogenetic relationships are depicted in Figure 2b.

Among host plants in this study were 14 gymnosperm species and 55 angiosperm species. Twenty‐six species of wood‐decay macrofungi were found on gymnosperm hosts, while 107 species were found on angiosperm hosts. We reconstructed phylogenetic trees for these four taxa using the same methods to facilitate further examination of the evolutionary adaptation of wood‐decay macrofungi to both gymnosperms and angiosperms. The four taxa are gymnosperms, angiosperms, wood‐decay macrofungi found on gymnosperm, and wood‐decay macrofungi found on angiosperms. These phylogenetic trees can be seen in Figures S1 and S2, respectively.

After examining the evolutionary adaptation relationship (Table 1), we found that evolutionary adaptation occurs of wood‐decay macrofungi to host plants (*p* = .012, *p* < .05). Evolutionary adaptation was observed of wood‐decay macrofungi to angiosperms (*p* = .025, *p* < .05). However, no evolutionary adaptation was observed of wood‐decay macrofungi to gymnosperms (*p* = .977).

**TABLE 1:** Results of the evolutionary adaptation test of wood‐decay macrofungi to host plants.

Category	*p*‐value	*Nperm*
All host plants and all wood‐decay macrofungi	0.012	999
Gymnosperms and their wood‐decay macrofungi	0.977	999
Angiosperms and their wood‐decay macrofungi	0.025	999

*Note*: The *p*‐value refers to the results of the ‘parafit’ test, with nperm indicating the number of repetitions.

### 3.2 The bipartite network structure between host plants and wood‐decay macrofungi

The relationship between wood‐decay macrofungi and angiosperms was found to be significantly influenced by evolutionary adaptation, while the relationship between gymnosperms and wood‐decay macrofungi was not. To explain this phenomenon, we established a bipartite network structure between host plants and their wood‐decay macrofungi (Figure S3).

**FIGURE 3:**
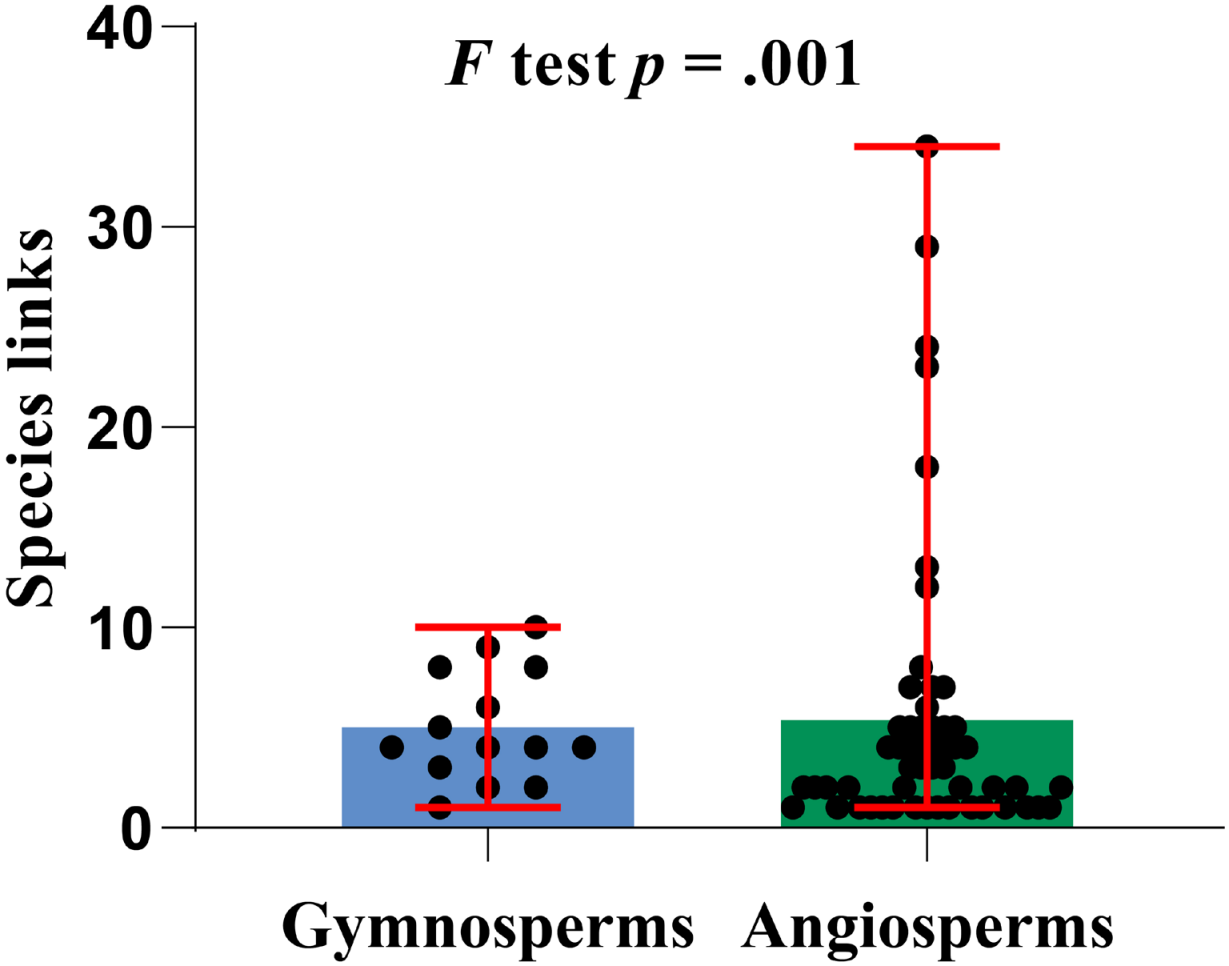
Numbers of links between wood‐decay macrofungi and their host plants, with results of *F*‐test shown. The red lines represent the mean standard deviations. The black dots represent each species, and the height of the box represents the average links.

In this study, we found 70 links between gymnosperms and wood‐decay macrofungi, with an average of 5.000 links per gymnosperm species (Table S1). In contrast, we found a total of 296 links between angiosperms and their wood‐decay macrofungi, with an average of 5.382 links per angiosperm species (Table S1). The average number of links between all wood‐decay macrofungi and all host plants was 5.304 (Table S1). We performed *F*‐tests on the number of links (Figure 3), with a *p*‐value of 0.001 (*p* < .05) for the *F*‐test, indicating significance. Thus, only minor variation was found in the number of links among gymnosperms and their wood‐decay macrofungi, indicating a more aggregated pattern, while more significant variation was found in the number of links among angiosperms and their wood‐decay macrofungi, indicating a more dispersed pattern overall.

Our calculation of the modularity coefficient is shown in Table 2, the highest modularity of gymnosperms and their wood‐decay macrofungi is 0.452, and the highest modularity of angiosperms and their wood‐decay macrofungi is 0.394. The modularity coefficients of gymnosperms and their wood‐decay macrofungi were always higher than the confidence interval (95%) obtained from null matrices, while the modularity coefficients of angiosperms and their wood‐decay macrofungi were always lower than the confidence interval (95%) obtained from null matrices. These results show that gymnosperms and their wood‐decay macrofungi are more modular, while angiosperms and their wood‐decay macrofungi are less stable. The modularity coefficient for angiosperms and their wood‐decay macrofungi was slightly lower (0.394). The modularity coefficient for gymnosperms and their wood‐decay macrofungi was higher (0.452). Thus, gymnosperms and their wood‐decay macrofungi had a higher modularity coefficient, indicating a higher degree of specialization between them, while angiosperms and their wood‐decay macrofungi had a lower modularity coefficient, indicating a lower degree of specialization between them. We also used the bipartite package to establish the bipartite network structure between wood‐decay macrofungi and their host plants (Figure S3), which allows for a more intuitive understanding of the connections between wood‐decay macrofungi and their host plants.

**TABLE 2:** Modularity between wood‐decay macrofungi and host plants.

Repetition	Gymnosperms and their wood‐decay macrofungi	Angiosperms and their wood‐decay macrofungi
1	0.452*	0.387
2	0.451*	0.382
3	0.449*	0.374
4	0.449*	0.394
5	0.452*	0.378

*Note*: The modularity ranges from 0 to 1, with higher values indicating more robust modularity and close specialization between plants and their wood‐decay macrofungi.

* Indicate significant network modularization at 95% confidence level.

### 3.3 Statistics of the growth quantity of wood‐decay macrofungi on gymnosperms and angiosperms

The differences in the occurrence of wood‐decay macrofungi on gymnosperms and angiosperms are as follows. Nine species of wood‐decay macrofungi were found exclusively on gymnosperms, 17 were found on both gymnosperms and angiosperms, and 90 were found only on angiosperms (Figure S4). Among the 26 species of wood‐decay macrofungi that were found on gymnosperms, 34.6% were exclusively associated with gymnosperms, while 65.4% were found on both gymnosperms and angiosperms (Figure S5). Among the 107 species of macrofungi found on angiosperms, 84.1% were found exclusively on angiosperms, while the remaining 15.9% were found on both gymnosperms and angiosperms (Figure S5).

## 4 DISCUSSION

In our study examining the evolutionary adaptation dynamics of 116 wood‐decay macrofungi to 69 host plants, we identified a significant evolutionary adaptation relationship. However, these relationships varied between angiosperms and gymnosperms. While a significant evolutionary adaptation link was established of wood‐decay macrofungi to angiosperms, such a relationship was not evident in gymnosperms. This differentiation highlights the differential evolutionary adaptation of wood‐decay macrofungi to angiosperms and gymnosperms.

### 4.1 The evolutionary adaptation of wood‐decay macrofungi to their host plants

Our study provides the first experimental evidence of a significant evolutionary adaptation of wood‐decay macrofungi to their host plants (*p* = .012, *p* < .05), supporting the long‐held belief in their interdependence (Cornelissen et al., 2023). This relationship suggests that closely related fungi tend to inhabit closely related plants, possibly due to the role of fungi in decomposing wood, which facilitates the forest ecosystem's nutrition and energy cycle (Li, Guo, et al., 2021; Li, Steenwyk, et al., 2021). Wood‐decay macrofungi decompose dead wood, making those complex compounds into simple nutrients (Floudas et al., 2012; Purahong et al., 2016). These simple nutrients are also easily ingested by plants (Hoppe et al., 2016). Therefore, after the appearance of wood‐decay macrofungi, its decomposition can promote nutrient cycling more effectively than burning. Our findings indicate that the accelerated nutrition cycle enhances plant growth conditions. The speed of nutrient cycling is faster so that plants can get nutrients faster during growth and development; therefore, the alternation of generations of plants is faster (Barrett & Willis, 2010). During this period, whenever a generation of plants dies, wood‐decay macrofungi will break down the dead wood. Therefore, this way indirectly increases the frequency of interactions between wood‐decay macrofungi and host plants. With the continuous promotion of this interaction, wood‐decay macrofungi and their host plants began to undergo evolutionary adaptation.

### 4.2 The evolutionary adaptation of wood‐decay macrofungi to gymnosperms differs from that to angiosperms

Our study examined the evolutionary adaptation of wood‐decay macrofungi to gymnosperms, as well as of wood‐decay macrofungi to angiosperms. We observed a significant evolutionary adaptation of wood‐decay macrofungi to angiosperms (*p* = .025, *p* < .05), which contrasted with the non‐significant relationship found in gymnosperms (*p* = .977). The possible reasons for these observations are discussed below.

Firstly, We calculated the modularity coefficients for both fungi‐plant interaction network structures (Table 2), and we found a relatively high modularity coefficient (0.452) for the gymnosperm‐macrofungi network. This high coefficient suggests a specialized, species‐specific interaction, implying a stable relationship that might not necessitate ongoing evolutionary adaptation (Beckett & Williams, 2013; Lutzoni et al., 2018). As a result, no detectable evolutionary adaptation phenomenon was observed of wood‐decay macrofungi to gymnosperms. In contrast, the modularity coefficient for angiosperms and their fungi was lower (0.394), indicating a less specialized relationship and pointing to more active evolutionary changes (Beckett & Williams, 2013; Lutzoni et al., 2018). Therefore, the relationship between wood‐decay macrofungi and angiosperms appears to be in a continuous state of evolutionary adaptation.

Our study also highlights the inconsistency in the evolutionary adaptation of wood‐decay macrofungi to their host plants. Figures S4– and S5 show that among the fungi found on gymnosperms, only 34.6% were exclusive to them, with the remaining 65.4% found on both gymnosperms and angiosperms. Conversely, among fungi on angiosperms, 84.1% were exclusive to angiosperms, and only 15.9% were common to both. This suggests that certain gymnosperm‐specific fungi might not have evolutionary adaptation with their hosts, potentially due to an inability to adapt to the evolving composition of gymnosperm wood. Consequently, the proportion of fungi exclusively decomposing gymnosperms has decreased. Meanwhile, fungi capable of decomposing both gymnosperms and the more decomposable angiosperms have become more dominant in gymnosperm decomposition. In angiosperms, the fungi that obligately decompose them are still predominant, evolving continuously in response to the angiosperms' evolution. As angiosperms are more decomposable than gymnosperms, wood‐decay macrofungi with strong decomposition abilities can also decompose angiosperms, accounting for a smaller proportion of the fungi found on angiosperm wood.

The evolutionary adaptation of wood‐decay macrofungi to gymnosperms differs markedly from that to angiosperms. This discrepancy can be attributed to several factors, discussed below. Firstly, gymnosperms boast a more extended evolutionary history compared to angiosperms (Pennisi, 2009). Gymnosperms and their associated wood‐decay macrofungi have undergone prolonged mutual selection processes (Lutzoni et al., 2018), leading to stable interaction patterns and fixed evolutionary adaptations. This long‐term relationship may explain why wood‐decay macrofungi to gymnosperms are less inclined toward evolutionary adaptation. Additionally, gymnosperms typically grow in specific soil environments to which they are well‐adapted (De La Torre et al., 2020), resulting in a relatively low frequency of interaction with wood‐decay macrofungi and, consequently, limited evolutionary potential. In contrast, angiosperms, which overtook gymnosperms as the dominant plants in the Late Cretaceous (Scott & Peter, 1988), have had a shorter duration of interaction with wood‐decay macrofungi. This shorter evolutionary timeframe means the stable, species‐specific relationships observed in gymnosperms may not have fully developed in angiosperms. Therefore, angiosperms and their wood‐decay macrofungi are still in the process of forming more stable, species‐specific relationships. In Purahong's research (Purahong et al., 2018), wood‐decay macrofungi colonizing gymnosperms showed less pronounced tree species preferences, The preference of wood rot macrofungi for angiosperms was obvious, which also verified our inference.

Furthermore, angiosperms are known for their faster and more frequent diversification processes (De La Torre et al., 2020; Moore et al., 2007; Qiu et al., 1999). Angiosperms have higher growth rates than gymnosperms, which enables quick adaptation to nutrient supplies, and the angiosperms facilitate soil nutrient release by producing easily decomposable litter (Berendse & Scheffer, 2009). Additionally, angiosperms have developed more complex root systems and adapted to a wider range of environments (Omary et al., 2022), leading to more frequent evolutionary changes. These factors collectively enhance the opportunities for angiosperms to interact with fungi (Floudas et al., 2012; Purahong et al., 2016), thereby increasing the potential for evolutionary adaptation of wood‐decay macrofungi to angiosperms. Considering that wood‐decay macrofungi likely originated around the same time as angiosperms (Lutzoni et al., 2018), it is plausible that there was an evolutionary adaptation of wood‐decay macrofungi to angiosperms. This evolutionary adaptation process reflects the dynamic and ongoing adaptation between these wood‐decay macrofungi and their changing environments.

## 5 CONCLUSIONS

In our study, we applied a host–parasite coevolution test model to investigate the relationships between wood‐decay macrofungi and their host plants. We found no significant evolutionary adaptation of these fungi to gymnosperms. However, a notable evolutionary adaptation relationship was evident of the fungi to angiosperms. This distinction is crucial for understanding forest carbon cycling and ecosystem sustainability. Our research also highlights an open question: does the evolutionary adaptation of these fungi to their host plants vary geographically? This remains an area for future exploration and discussion.

## AUTHOR CONTRIBUTIONS

**Xuetong Zhang:** Conceptualization (equal); data curation (equal); investigation (lead); methodology (equal); resources (equal); validation (equal); visualization (equal); writing – original draft (lead). **Yuran Dong:** Conceptualization (equal); data curation (equal); formal analysis (equal); methodology (equal); software (equal); validation (equal); visualization (equal); writing – original draft (supporting); writing – review and editing (equal). **Yuying Li:** Data curation (equal); formal analysis (equal); investigation (lead); methodology (equal); resources (equal); software (equal); visualization (equal); writing – review and editing (equal). **Xiuping Wu:** Conceptualization (supporting); data curation (equal); formal analysis (equal); investigation (equal); resources (equal); validation (equal); visualization (equal). **Siyu Chen:** Data curation (equal); formal analysis (equal); investigation (equal); methodology (equal); resources (equal); validation (supporting); visualization (supporting). **Mingyuan Wang:** Conceptualization (supporting); data curation (equal); formal analysis (equal); investigation (equal); resources (supporting); visualization (equal). **Yao Li:** Conceptualization (equal); data curation (equal); formal analysis (supporting); investigation (equal); resources (equal); supervision (supporting). **Zhiwei Ge:** Conceptualization (supporting); data curation (equal); investigation (equal); resources (equal); writing – review and editing (supporting). **Min Zhang:** Conceptualization (supporting); data curation (equal); investigation (supporting); methodology (equal); validation (supporting). **Lingfeng Mao:** Conceptualization (equal); formal analysis (equal); methodology (equal); project administration (equal); resources (equal); validation (supporting); visualization (supporting); writing – review and editing (equal).

## CONFLICT OF INTEREST STATEMENT

The authors declare no conflict of interest.

## Supporting information


Appendix S1.

Appendix S2.

## Data Availability

The data that supports the findings of this study are available in the supplementary material of this article.
