# Supplementary material for: The evolutionary adaptation of wood‐decay macrofungi to host gymnosperms differs from that to host angiosperms
Source: Ecol Evol. 2024 Jul 17;14(7):e70019. doi: 10.1002/ece3.70019 (PMC11255378; doi:10.1002/ece3.70019)
Supplement: Supplementary file 2 — Appendix S2. [file ECE3-14-e70019-s001.docx]

Figure S1-S5


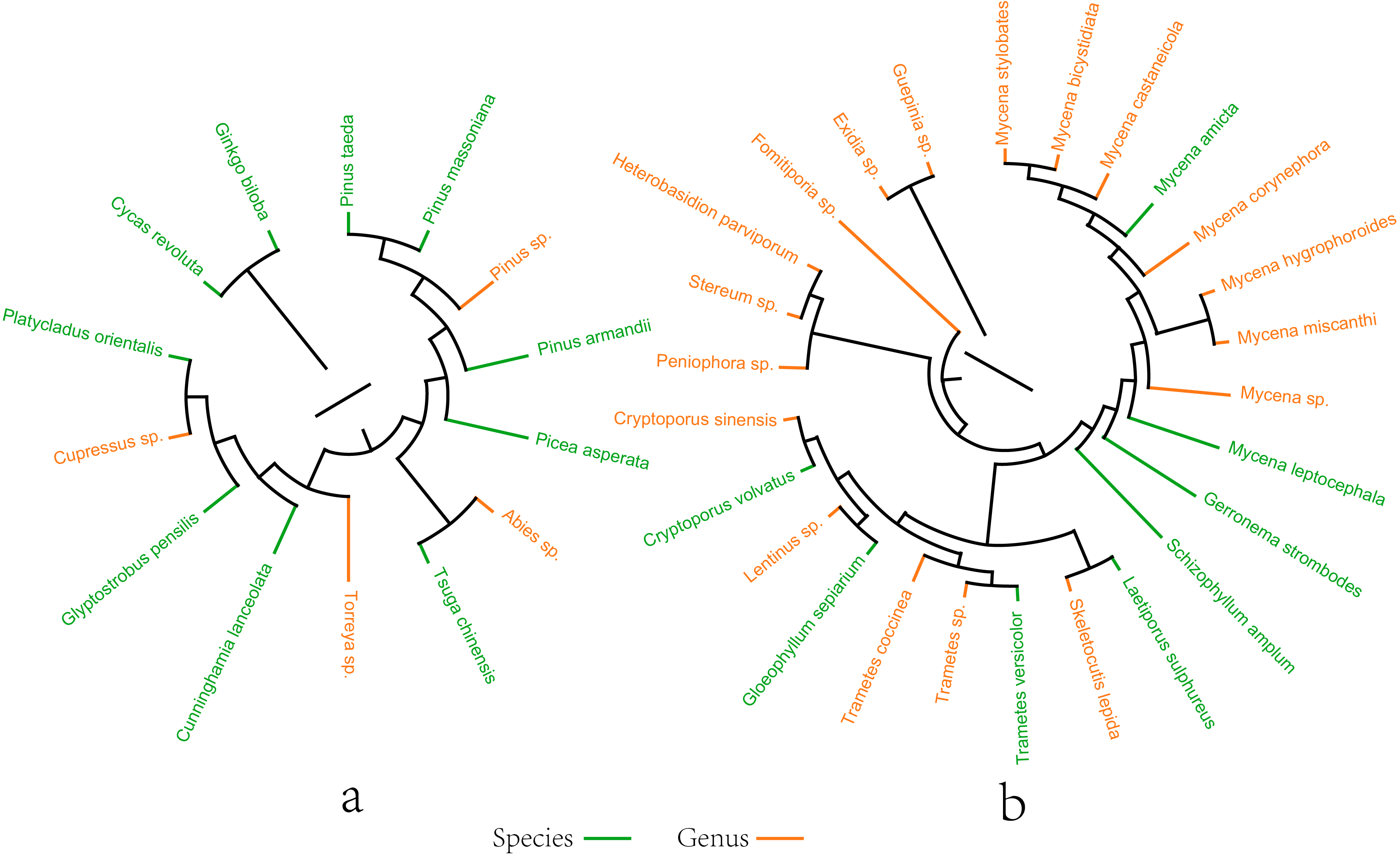
Figure S1 Phylogenetic tree of gymnosperms (a) and their associated wood-decay macrofungi (b). The green samples were identified at the species level, The yellow samples were identified at the genus level.


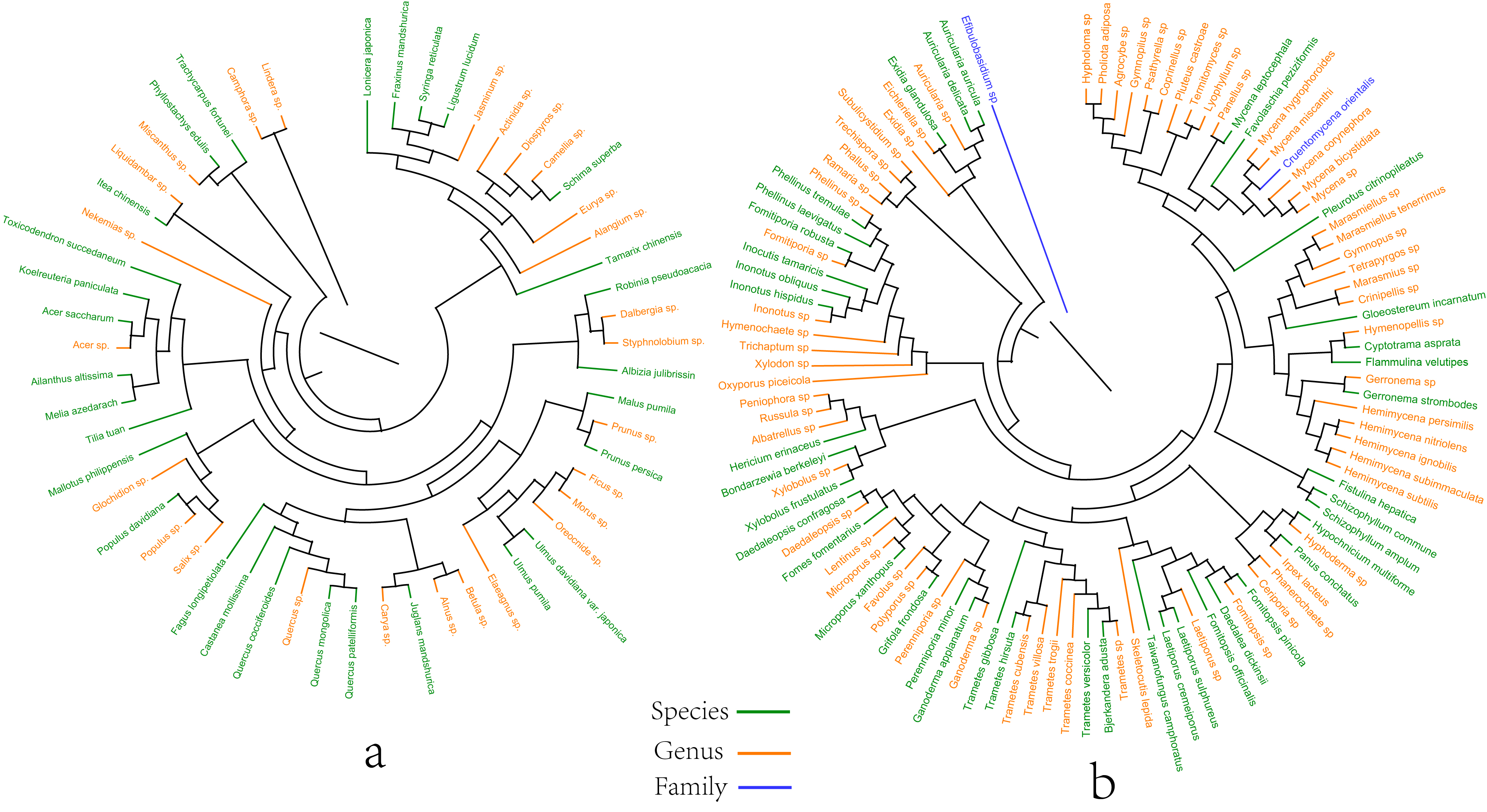


Figure S2 Phylogenetic tree of angiosperms (a) and their associated wood-decay macrofungi (b). The green samples were identified at the species level, The yellow samples were identified at the genus level, and The blue samples were identified at the family level.


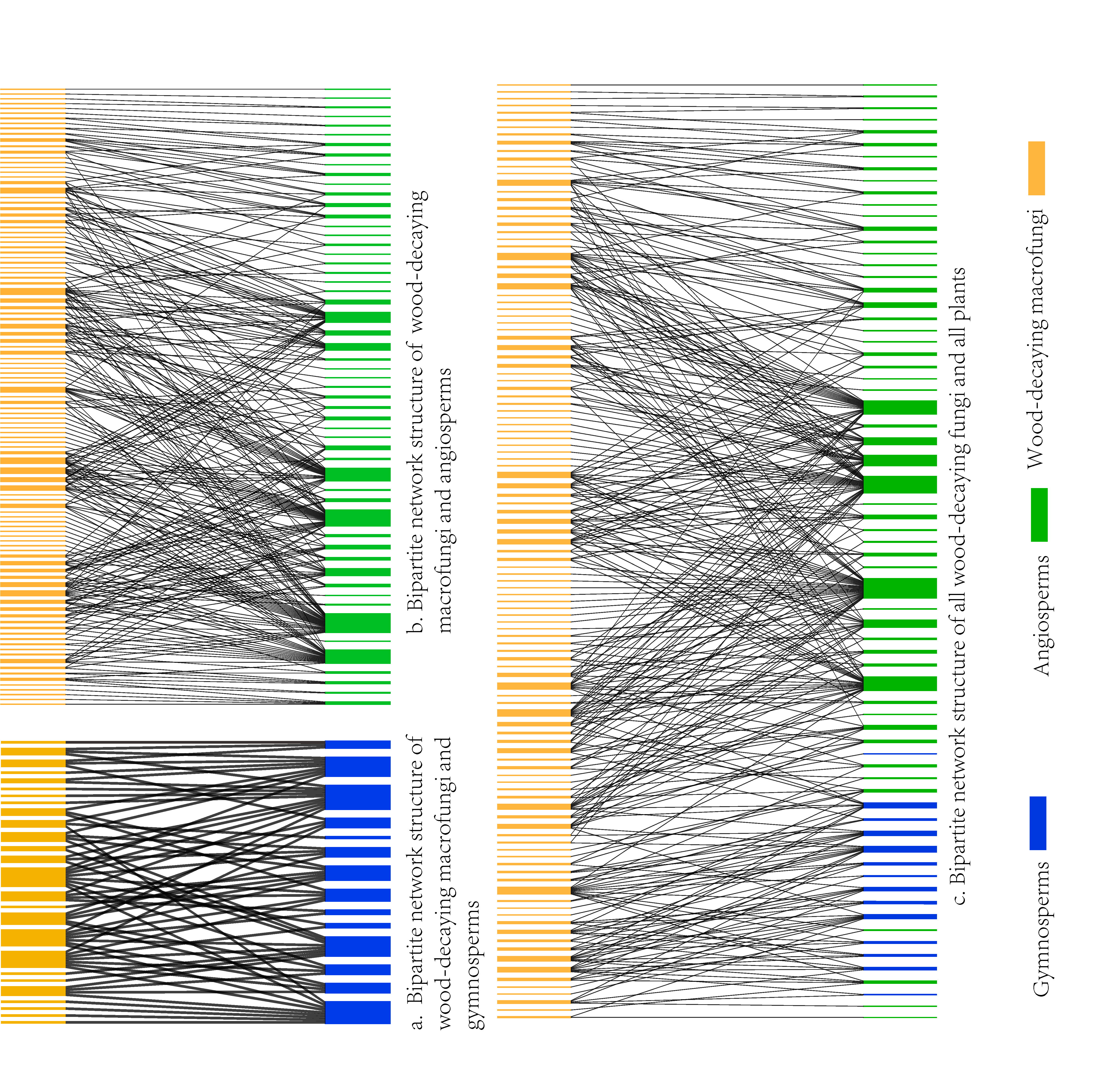


Figure S3 The bipartite network structure between wood-decay macrofungi and their host plants. The blue modules are gymnosperms, the green modules are angiosperms, and the yellow modules are wood-decay macrofungi


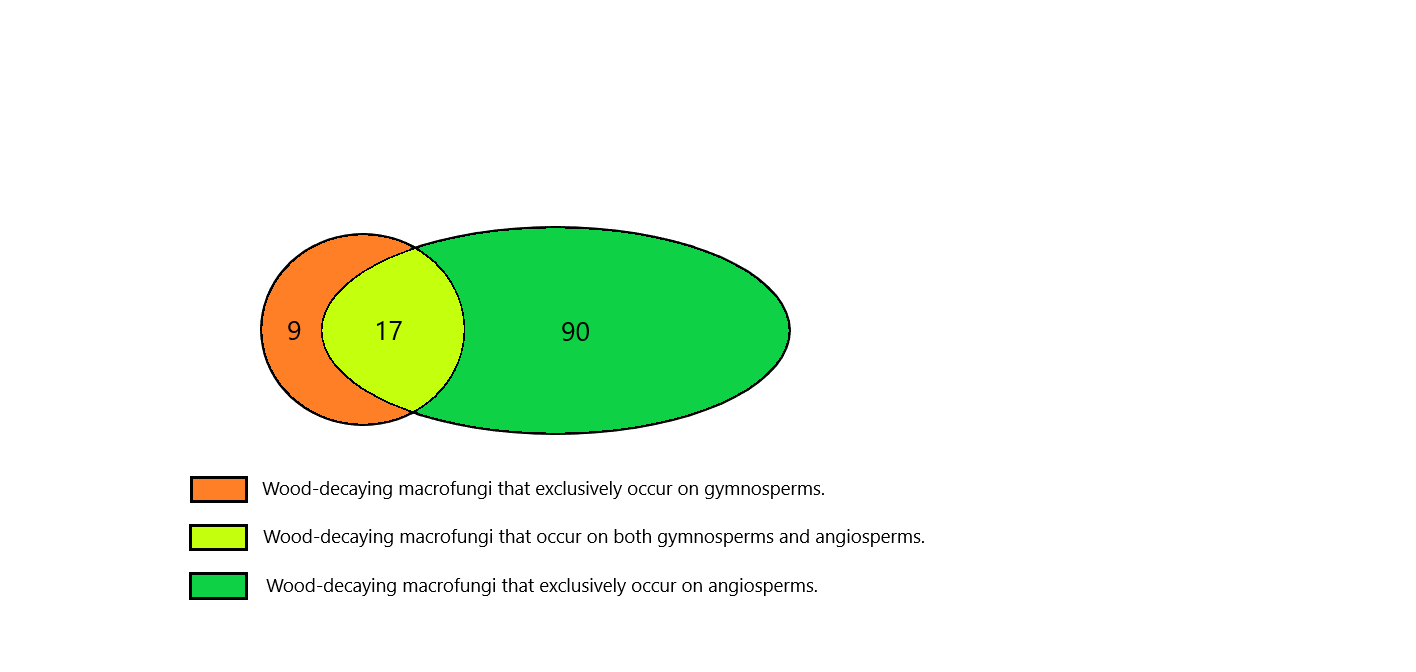


Figure S4 The numbers of wood-decay macrofungi found growing on different plant types


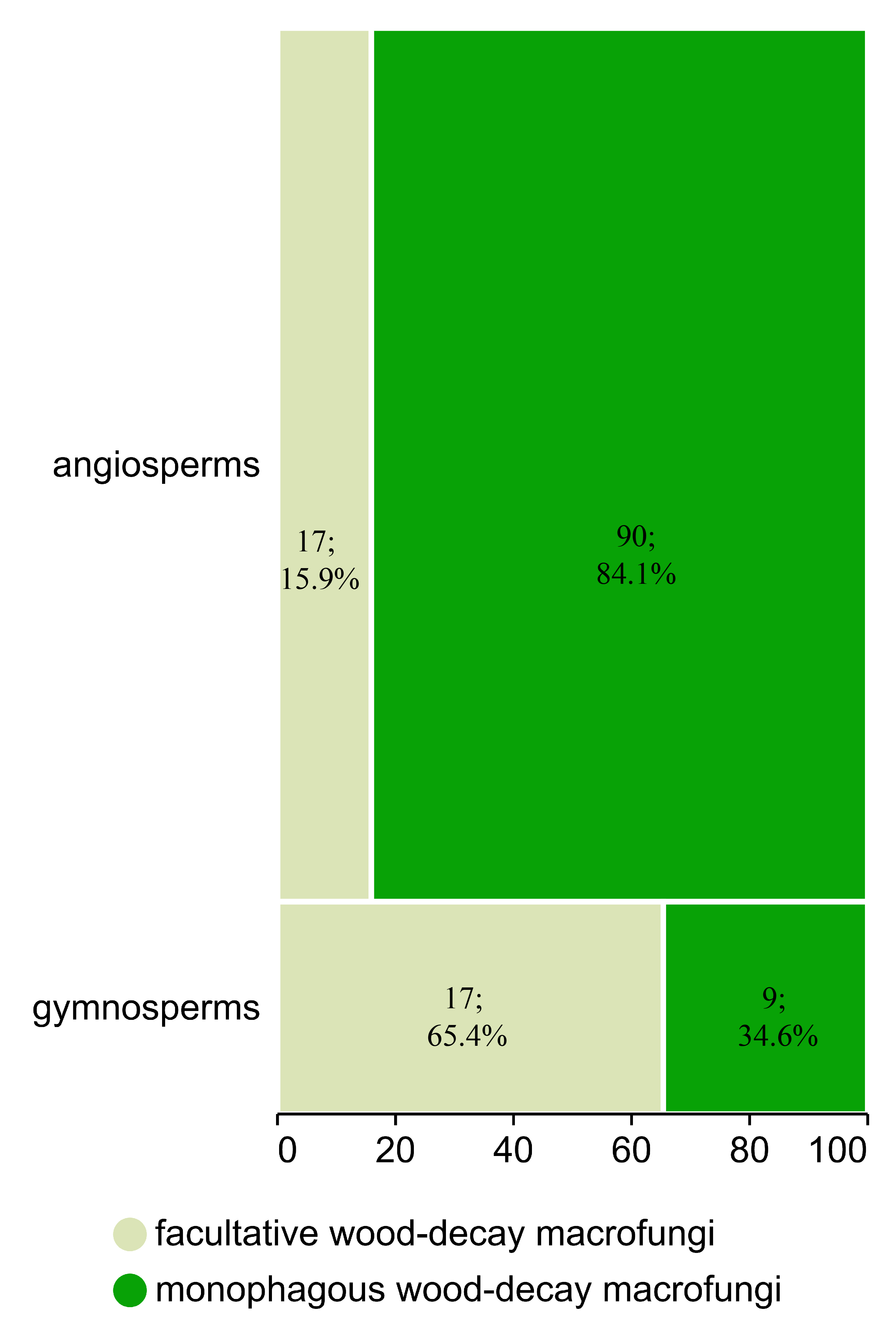


Figure S5 The proportion of different types of wood-decay macrofungi found growing on gymnosperms and angiosperms

Table S1

**Table S1** Average number of links between wood-decay macrofungi and host plants.

| Category | average links |
| --- | --- |
| Gymnosperms and their wood-decay macrofungi | 5.000 |
| Angiosperms and their wood-decay macrofungi | 5.382 |
| All host plants and all wood-decay macrofungi | 5.304 |

The average links refers to the number of matches between fungi and plants.
